# Phelan-McDermid syndrome: a review of the literature and practice parameters for medical assessment and monitoring

**DOI:** 10.1186/1866-1955-6-39

**Published:** 2014-10-08

**Authors:** Alexander Kolevzon, Benjamin Angarita, Lauren Bush, A Ting Wang, Yitzchak Frank, Amy Yang, Robert Rapaport, Jeffrey Saland, Shubhika Srivastava, Cristina Farrell, Lisa J Edelmann, Joseph D Buxbaum

**Affiliations:** Seaver Autism Center for Research and Treatment, Icahn School of Medicine at Mount Sinai, One Gustave L. Levy Place, New York, NY 10029 USA; Department of Psychiatry, Icahn School of Medicine at Mount Sinai, One Gustave L. Levy Place, New York, NY 10029 USA; Department of Pediatrics, Icahn School of Medicine at Mount Sinai, One Gustave L. Levy Place, New York, NY 10029 USA; Department of Neuroscience, Icahn School of Medicine at Mount Sinai, One Gustave L. Levy Place, New York, NY 10029 USA; Department of Neurology, Icahn School of Medicine at Mount Sinai, One Gustave L. Levy Place, New York, NY 10029 USA; Department of Genetics and Genomic Sciences, Icahn School of Medicine at Mount Sinai, One Gustave L. Levy Place, New York, NY 10029 USA; Division of Endocrinology and Diabetes, Icahn School of Medicine at Mount Sinai, One Gustave L. Levy Place, New York, NY 10029 USA; Department of Cardiology, Icahn School of Medicine at Mount Sinai, One Gustave L. Levy Place, New York, NY 10029 USA; Division of Behavioral Pediatrics, Icahn School of Medicine at Mount Sinai, One Gustave L. Levy Place, New York, NY 10029 USA; Friedman Brain Institute, Icahn School of Medicine at Mount Sinai, One Gustave L. Levy Place, New York, NY 10029 USA; Mindich Child Health and Development Institute, Icahn School of Medicine at Mount Sinai, One Gustave L. Levy Place, New York, NY 10029 USA

**Keywords:** Phelan-McDermid syndrome, 22q13 deletion syndrome, *SHANK3*, Autism, Autism spectrum disorder, Neurodevelopmental disorders, Practice parameters

## Abstract

Autism spectrum disorder (ASD) and intellectual disability (ID) can be caused by mutations in a large number of genes. One example is *SHANK3* on the terminal end of chromosome 22q. Loss of one functional copy of *SHANK3* results in 22q13 deletion syndrome or Phelan-McDermid syndrome (PMS) and causes a monogenic form of ASD and/or ID with a frequency of 0.5% to 2% of cases. *SHANK3* is the critical gene in this syndrome, and its loss results in disruption of synaptic function. With chromosomal microarray analyses now a standard of care in the assessment of ASD and developmental delay, and with the emergence of whole exome and whole genome sequencing in this context, identification of PMS in routine clinical settings will increase significantly. However, PMS remains a rare disorder, and the majority of physicians have never seen a case. While there is agreement about core deficits of PMS, there have been no established parameters to guide evaluation and medical monitoring of the syndrome. Evaluations must include a thorough history and physical and dysmorphology examination. Neurological deficits, including the presence of seizures and structural brain abnormalities should be assessed as well as motor deficits. Endocrine, renal, cardiac, and gastrointestinal problems all require assessment and monitoring in addition to the risk of recurring infections, dental and vision problems, and lymphedema. Finally, all patients should have cognitive, behavioral, and ASD evaluations. The objective of this paper is to address this gap in the literature and establish recommendations to assess the medical, genetic, and neurological features of PMS.

## Review

### Introduction

Gene discovery approaches, followed by functional analysis of model systems, have elucidated the neurobiology of several genetic subtypes of autism spectrum disorder (ASD). ASD can now be conceived of as having many distinct genetic risk genes and one example is *SHANK3* on terminal chromosome 22q. Studies indicate that loss of one functional copy (haploinsufficiency) of *SHANK3* through deletion or mutation causes a monogenic form of ASD with a frequency of at least 0.5% of ASD cases [1–7]. The rate of *SHANK3* loss in children with moderate to profound intellectual disability (ID) appears to be up to 2% [1, 6]. *SHANK3* is the critical gene in this syndrome [2, 8], and its loss is sufficient to cause Phelan-McDermid syndrome (PMS; OMIM ID 606232). *SHANK3* encodes a scaffolding protein in the postsynaptic density of glutamatergic synapses and is known to play a critical role in synaptic function [9]. Although *SHANK3* deletions and mutations account for a relatively small proportion of ASD and ID cases, recent evidence suggests that many different genetic causes of ASD and ID converge on several common pathways, including the *SHANK3* pathway [10, 11]. Using *Shank3*-deficient mice, specific deficits in synaptic function and plasticity in glutamatergic signaling have been documented [12–17]. Interestingly, reversal of synaptic deficits has been shown with an experimental therapeutic (insulin-like growth factor-1; IGF-1) in mouse [18] and human neuronal models [19]. With chromosomal microarray now recommended as a standard of care for the assessment of ASD [20–23], it is expected that identification of PMS will increase significantly in routine clinical settings. To date, about 1,200 cases have been identified worldwide according to the Phelan-McDermid Syndrome Foundation, yet few physicians have encountered the syndrome. While some clinical guidelines exist [24–28], comprehensive medical assessment parameters have yet to be developed.

Clinically, there have been at least 13 case series published in the literature that describe approximately 584 affected individuals (see Tables 1 and 2), although several studies present overlapping cases and the majority of data has been collected using parent surveys [27, 29–40]. Reports highlight a broad and clinically heterogeneous phenotype; clinical features consistently reported include global developmental delay, absent or severely delayed speech, autistic features, minor dysmorphic features, and hypotonia. Affected individuals are more likely to suffer from medical complications, such as gastrointestinal disease, renal abnormalities, upper respiratory tract infections, and seizures. Brain MRI studies suggest a higher than expected prevalence of arachnoid cysts, ventriculomegaly, dysmyelination, and morphological changes of the corpus callosum [27, 30, 36, 37, 41]. Recently, several case reports have also proposed a putative association between PMS and atypical bipolar disorder and progressive loss of skills during adolescence or adulthood [29, 42, 43]. Comprehensive mapping of the phenotype using systematic and prospective assessment was recently reported in a sample of 32 subjects highlighting the high prevalence of ASD in these patients, at 84% [37].

**Table 1:** **Dysmorphic features associated with PMS**

Dysmorphic feature	Nesslinger et al. [ [35] ]	Phelan et al. [ [27] ] ^a^	Luciani et al. [ [33] ]	Manning et al. [ [34] ]	Koolen et al. [ [32] ]	Jeffries et al. [ [31] ]	Cusmano-Ozog et al. [ [41] ] ^a^	Dhar et al. [ [30] ]	Rollins [ [38] ] ^a^	Denayer et al. [ [29] ]	Sarasua et al. [ [40] ] ^a^	Soorya et al. [ [37] ]	Sarasua et al. [ [39] ] ^a^	Average (%)
	***n*** = 7	***n*** = 37	***n*** = 33	***n*** = 11	***n*** = 9	***n*** = 30	***n*** = 107	***n*** = 13	***n*** = 43	***n*** = 7	***n*** = 54	***n*** = 32	***n*** = 201	
Sparse hair/abnormal whorl	n/a	n/a	n/a	n/a	n/a	n/a	n/a	n/a	n/a	n/a	n/a	16% (5/32)	n/a	16
Macrocephaly	n/a	n/a	n/a	n/a	n/a	7% (2/30)	n/a	n/a	21% (9/43)	n/a	23% (11/47)	31% (10/32)	18% (20/110)	17
Microcephaly	n/a	n/a	n/a	n/a	n/a	13% (4/30)	n/a	n/a	11% (5/43)	n/a	14% (6/42)	6% (2/32)	11% (12/110)	12
Dolichocephaly	86% (6/7)	57% (21/37)	n/a	n/a	0% (0/9)	23% (7/30)	30% (32/107)	n/a	n/a	n/a	30% (16/54)	25% (8/32)	32% (36/113)	37
Periorbital fullness	n/a	n/a	n/a	45% (5/11)	n/a	60% (18/30)	n/a	n/a	n/a	n/a	n/a	25% (8/32)	55% (60/109)	46
Epicanthal folds	57% (4/7)	41% (15/37)	n/a	73% (8/11)	n/a	37% (11/30)	30% (32/107)	70% (9/13)	n/a	n/a	n/a	31% (10/32)	47% (52/111)	48
Ptosis	43% (3/7)	57% (21/37)	n/a	n/a	0%	n/a	23% (25/107)	n/a	n/a	n/a	n/a	3% (1/32)	47% (53/112)	29
Deep set eyes	n/a	n/a	n/a	n/a	n/a	n/a	n/a	n/a	n/a	14% (1/7)	n/a	6% (2/32)	31% (34/111)	19
Long eyelashes	n/a	n/a	n/a	45% (5/11)	n/a	37% (11/30)	n/a	n/a	n/a	n/a	n/a	44% (14/32)	93% (105/113)	58
Hypertelorism	n/a	n/a	n/a	36% (4/11)	n/a	17% (5/30)	n/a	n/a	n/a	n/a	n/a	13% (4/32)	n/a	22
Wide nasal bridge	n/a	n/a	n/a	n/a	n/a	n/a	n/a	n/a	n/a	n/a	n/a	16% (5/32)	n/a	16
Bulbous nose	n/a	n/a	n/a	n/a	n/a	80% (24/30)	n/a	70% (9/13)	n/a	n/a	61% (33/54)	47% (15/32)	n/a	65
Low set ears	n/a	n/a	n/a	n/a	n/a	7% (2/30)	n/a	n/a	n/a	14% (1/7)	n/a	3% (1/32)	n/a	5
Ear anomalies	86% (6/7)	65% (24/37)	82% (27/33)	27% (3/11)	67% (6/9)	73% (22/30)	54% (58/107)	70% (9/13)	n/a	n/a	n/a	41% (13/32)	n/a	63
Full lips	n/a	n/a	n/a	n/a	n/a	n/a	n/a	n/a	n/a	n/a	n/a	31% (10/32)	n/a	31
High arched palate	n/a	n/a	n/a	n/a	n/a	n/a	n/a	n/a	n/a	n/a	n/a	25% (8/32)	47% (49/104)	36
Long philtrum	n/a	n/a	n/a	n/a	n/a	n/a	n/a	n/a	n/a	n/a	n/a	16% (5/32)	n/a	16
Malocclusion/widely spaced teeth	n/a	n/a	n/a	n/a	n/a	n/a	n/a	n/a	n/a	n/a	n/a	19% (6/32)	n/a	19
Micrognathia	n/a	n/a	n/a	n/a	n/a	10% (3/30)	n/a	n/a	n/a	n/a	n/a	13% (4/32)	n/a	12
Full cheeks	n/a	n/a	n/a	n/a	n/a	n/a	n/a	n/a	n/a	n/a	n/a	25% (8/32)	n/a	25
Malar hypoplasia	n/a	n/a	n/a	n/a	n/a	n/a	n/a	n/a	n/a	n/a	n/a	9% (3/32)	n/a	9
Flat midface	n/a	n/a	n/a	n/a	n/a	n/a	n/a	n/a	n/a	n/a	n/a	3% (1/32)	n/a	3
Pointed chin	n/a	62% (23/37)	n/a	n/a	56% (5/9)	n/a	27% (29/107)	n/a	n/a	n/a	n/a	22% (7/32)	52% (58/111)	44
Fifth finger clinodactyly	n/a	14% (5/37)	n/a	n/a	n/a	n/a	n/a	n/a	n/a	n/a	n/a	9% (3/32)	n/a	12
Large fleshy hands	n/a	68% (25/37)	n/a	n/a	56% (5/9)	47% (14/30)	33% (35/107)	46% (6/13)	n/a	n/a	55% (29/53)	53% (17/32)	63% (71/112)	53
Hypoplastic/dysplastic nails	n/a	78% (29/37)	n/a	27% (3/11)	56% (5/9)	3% (1/30)	36% (39/107)	23% (3/13)	n/a	n/a	75% (40/53)	34% (11/32)	73% (81/111)	45
Hyper-extensibility	n/a	n/a	n/a	n/a	n/a	n/a	n/a	n/a	n/a	n/a	n/a	25% (8/32)	61% (68/111)	86
Abnormal spine curvature	n/a	n/a	n/a	n/a	n/a	n/a	n/a	n/a	n/a	n/a	n/a	22% (7/32)	n/a	22
Sacral dimple	n/a	n/a	n/a	n/a	n/a	n/a	n/a	n/a	n/a	n/a	37% (19/52)	13% (4/32)	n/a	25
Syndactyly of toes 2 and 3	n/a	38% (14/37)	n/a	45% (5/11)	n/a	43% (13/30)	23% (25/107)	n/a	n/a	n/a	n/a	9% (3/32)	48% (53/110)	34

n/a = not available.

^a^These studies included cases previously reported in the literature and their samples overlap, at least partially.

**Table 2:** **Medical features associated with PMS**

Medical feature	Nesslinger et al. [ [35] ]	Phelan et al. [ [27] ] ^a^	Luciani et al. [ [33] ]	Manning et al. [ [34] ]	Koolen et al. [ [32] ]	Jeffries et al. [ [31] ]	Cusmano-Ozog et al. [ [41] ] ^a^	Dhar et al. [ [30] ]	Rollins [ [38] ] ^a^	Denayer et al. [ [29] ]	Sarasua et al. [ [40] ] ^a^	Soorya et al. [ [37] ]	Sarasua et al. [ [39] ] ^a^	Average (%)
	***n*** = 7	***n*** = 37	***n*** = 33	***n*** = 11	***n*** = 9	***n*** = 30	***n*** = 107	***n*** =13	***n*** = 43	***n*** = 7	***n*** = 54	***n*** = 32	***n*** = 201	
Hypotonia	100% (7/7)	97% (36/37)	82% (27/33)	82% (9/11)	89% (8/9)	47% (14/30)	86% (92/107)	31% (4/13)	n/a	29% (2/7)	80% (48/60)	75% (24/32)	75% (82/110)	73
Sleep disturbance	n/a	n/a	n/a	n/a	n/a	n/a	n/a	n/a	n/a	n/a	n/a	41% (13/32)	46% (12/26)	44
Gastroesophageal reflux	n/a	n/a	n/a	n/a	n/a	n/a	n/a	n/a	n/a	n/a	n/a	44% (14/32)	42% (62/149)	43
Increased pain tolerance	n/a	n/a	n/a	0%	n/a	10% (3/30)	31% (33/107)	n/a	n/a	n/a	n/a	88% (28/32)	77% (131/170)	42
Constipation/diarrhea	n/a	n/a	n/a	n/a	n/a	n/a	n/a	n/a	n/a	n/a	n/a	38% (12/32)	41% (11/27)	40
Brain imaging abnormalities	14% (1/7)	n/a	n/a	9% (2/23)	n/a	7% (2/30)	n/a	56% (5/9)	n/a	43% (3/7)	n/a	75% (21/28)	19% (24/129)	32
Recurring upper respiratory tract infections	n/a	n/a	n/a	n/a	n/a	n/a	n/a	8% (1/13)	n/a	n/a	n/a	53% (17/32)	n/a	30
Renal abnormalities	n/a	n/a	n/a	n/a	n/a	17% (5/30)	n/a	n/a	n/a	n/a	n/a	38% (12/32)	26% (39/148)	27
Lymphedema	29% (2/7)	n/a	n/a	n/a	n/a	n/a	n/a	23% (3/13)	n/a	n/a	n/a	22% (7/32)	24% (26/108)	25
Seizures (febrile and/or non-febrile)	14% (1/7)	27% (10/37)	24% (8/33)	27% (3/11)	n/a	17% (5/30)	23% (25/107)	31% (4/13)	n/a	14% (1/7)	19% (10/54)	41% (13/32)	27% (41/151)	24
Esotropia/strabismus	n/a	n/a	n/a	n/a	n/a	13% (4/30)	n/a	n/a	n/a	14% (1/7)	25% (13/53)	6% (2/32)	26% (29/109)	17
Short stature/delayed growth	0% (0/7)	n/a	12% (4/33)	n/a	n/a	n/a	11% (12/107)	n/a	12% (5/43)	n/a	13% (5/40)	13% (4/32)	11% (11/96)	12
Tall stature/accelerated growth	14% (1/7)	n/a	18% (6/33)	n/a	n/a	7% (2/30)	n/a	n/a	11% (5/43)	n/a	13% (5/40)	3% (1/32)	9% (9/96)	11
Cardiac defects	n/a	n/a	n/a	n/a	n/a	13% (4/30)	n/a	n/a	n/a	n/a	n/a	3% (1/32)	n/a	8
Precocious or delayed puberty	n/a	n/a	n/a	n/a	n/a	n/a	n/a	n/a	n/a	n/a	n/a	0% (0/32)	12% (15/121)	6
Hypothyroidism	n/a	n/a	n/a	n/a	n/a	n/a	n/a	n/a	n/a	n/a	n/a	3% (1/32)	6% (7/121)	5

n/a = not available.

^a^These studies included cases previously reported in the literature and their samples overlap, at least partial.

Several studies have also examined genotype-phenotype correlations with conflicting results. [31–33, 35, 37, 39, 40, 44, 45]. Despite small sample sizes and methodological challenges, correlations have been observed between larger deletion sizes and minor dysmorphic features [31, 37, 44], number of medical comorbidities [37], the Developmental Profile [45, 46], hypotonia [33, 44], and the absence of an ASD diagnosis [44]. In contrast to the latter study, another study found that larger deletion sizes were associated with more severe social-communication impairments related to ASD [37], whereas others have found no association between deletion size and phenotypic severity [32, 35].

While there is agreement about core deficits of PMS, there have been no established parameters to guide evaluation and medical monitoring of the syndrome. The objective of this paper is to make recommendations to assess the medical, genetic, and neurological features of PMS.

### Clinical genetics

Genetic testing is necessary to confirm the presence of deletions or mutations in *SHANK3*. Chromosomal microarray analysis (CMA) should be done as a first-tier work-up for any child with ASD or developmental delays [20–23]. CMA will ascertain the majority of PMS cases which most often result from deletions or other structural rearrangements resulting in copy number loss of varying size on 22q13 but will not identify patients with pathogenic *SHANK3* variants or smaller intragenic deletions or duplications which disrupt gene function [30]. Deletions at 22q13 occur *de novo* in the majority of patients although in 20% of cases, a parent carries a balanced rearrangement [45], hence there is significant risk of recurrence in some families. For this reason, chromosome analysis should be performed in conjunction with positive CMA findings to explore the presence of ring chromosome 22 and translocations. Biological parents should be tested with fluorescence *in situ* hybridization (FISH) to rule out a translocation or inversion in order to determine heritability and risk of recurrence within families. A balanced translocation or inversion involving chromosome 22 in a parent significantly increases the risk of recurrence in families and siblings of the proband should be tested when relevant [47–50]. There have been several reports of probands with apparently *de novo* deletions with siblings with identical deletions, likely resulting from germline mosaicism in a parent [4, 42, 51]. Thus, although the recurrence risk for future pregnancies is low for apparently *de novo* deletions, it is marginally greater than in the general population because parents may have germline mosaicism. Finally, for cases where PMS is part of the differential diagnosis, Sanger or next generation sequencing should be used to test for *SHANK3* mutations if CMA and karyotyping are unrevealing. In addition, it should be noted that several clinical laboratories offer autism sequencing panels which include the *SHANK3* gene. In this situation, it is important to determine whether the clinical laboratory offers complete sequencing of the *SHANK3* gene. Indeed, *SHANK3* is one of the most GC-rich genes in the genome, and reliable sequencing requires considerable optimization. Supplemental testing by multiplex ligation-dependent probe amplification (MLPA) or other dosage-sensitive methods for detection of smaller intragenic deletions and duplications below the resolution of CMA may be considered as some clinical laboratories will include these after a negative sequencing result.

**Figure 1:**
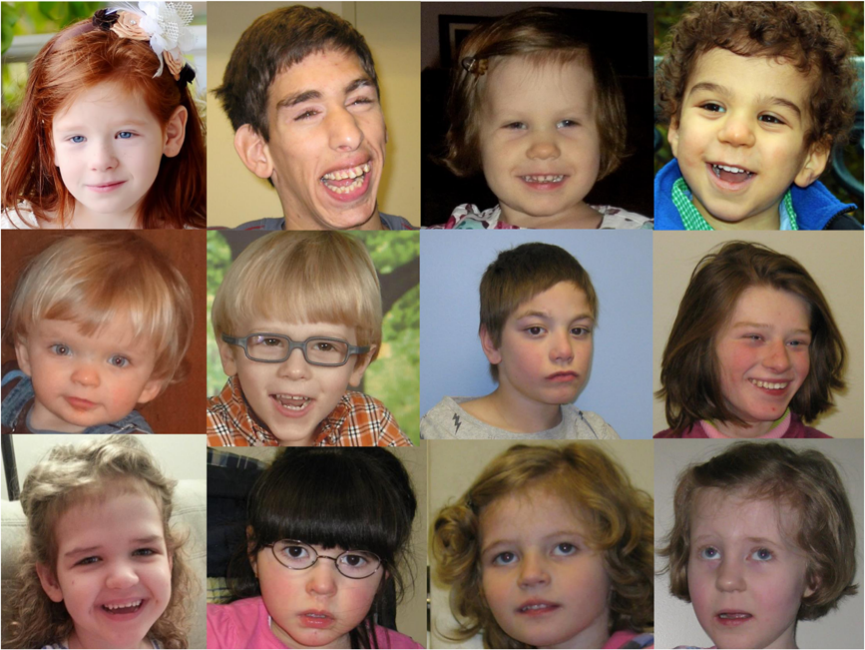
**Images of individuals with Phelan-McDermid syndrome illustrating common dysmorphic facial features, including long eyelashes, bulbous nose, and pointed chin.** All images are provided with guardian consent.

Clinical genetics evaluations and dysmorphology exams should be performed by a clinical geneticist to assess growth, pubertal development, head size, craniofacial features, digits, extremities, chest, spine, skin, and screen for organ malformations (such as congenital heart or renal defects). Most patients with PMS have at least one dysmorphic feature, although none are specific. The most common features are large fleshy hands, long eyelashes, pointed chin, prominent/dysplastic ears, bulbous nose, full lips, hypoplastic/dysplastic nails, and dolichocephaly (see Figure 1 and Table 1). All images are provided with guardian consent.

### Cognitive/behavioral assessment

All patients with PMS should be referred to centers with expertise in developmental disorders for comprehensive cognitive, behavioral, and ASD evaluations. To date, clinical methods to assess the prevalence of ASD in PMS have varied significantly. Studies that prospectively evaluate ASD suggest rates from 0% (0/8) [36], 44% (12/27) [31], 60% (3/5) [30], 84% (27/32) [37] to 94% (17/18) [27]. Of these studies, only Soorya and colleagues [37] used gold-standard diagnostic instruments—the Autism Diagnostic Interview-Revised and the Autism Diagnostic Observation Schedule. Given the limitations of ASD-specific diagnostic tools in individuals with significant ID, integrating careful clinical evaluation, caregiver reports, and structured direct observation is necessary in PMS. Cognitive assessments must likewise use standardized instruments appropriate for individuals with significant language delays and intellectual disability. Referrals for speech and language therapy, physical therapy, and occupational therapy evaluations should also be made. A diagnosis of ASD may aid in designing individualized educational and related service treatment plans and in justifying services through the board of education and insurance providers. Therapies should be delivered early and intensively given the severity of disability in many affected children. Because of significant learning and attention deficits in these patients, increased frequency of treatment with shorter duration of sessions may be favorable. While the cognitive and behavioral assessment of affected individuals is critical, it is nevertheless considered outside of the scope of this paper and will be addressed in subsequent recommendations.

### Neurology

Neurological examinations should be conducted to evaluate gross motor skills and gait, fine motor coordination, cranial nerves, and deep tendon reflexes. In PMS, evidence supports the high prevalence of hypotonia (see Table 2), delays in achieving motor milestones, and inability to ambulate, among other motor deficits [27, 30–34, 36, 37]. Feeding difficulties are a common early sign, possibly associated with hypotonia. Gait is almost uniformly affected in PMS, and abnormalities include steppage gait, toe walking, and variable degrees of broad-based ataxic gait. Careful assessment of this domain is important, as is appropriate referrals for pediatric physiatry, physical therapy, and orthopedics to explore the possibility of various interventions, including orthotics and bracing. Abnormality of posture, motor coordination and motor planning, though not specific to PMS, are almost invariably seen. There is also an increased risk of scoliosis. Given the broad array of motor deficits, low muscle tone and related feeding problems, physical, occupational, and feeding therapy should be initiated as early as possible. Screening for neuromuscular scoliosis should be performed at every routine visit. Head growth may also be abnormal, and some patients have macrocephaly (see Table 1); head circumference measurements should be performed routinely in PMS up to age 36 months.

#### Seizure assessment

Neurological phenotypic features are in need of further clarification in PMS, and few studies have reported electroencephalography (EEG) results. Most descriptions of PMS report higher than expected rates of seizure disorders; however, recording methods are not consistent and findings have not been well replicated. EEG recordings have not been prospectively collected in published studies of PMS, although existing case series report on the prevalence of seizure disorders using retrospective review and parent survey methods [27, 30, 31, 33, 36, 37, 39]. Prevalence estimates of seizure disorders range from 0% (0/8) [36] to 31% (4/13) [30], depending on the study. In a recent study, 41% (13/32) of participants had clinical seizures reported by parents, including 22% (7/32) with febrile seizures only, one participant with complex febrile seizures, and another patient who required temporal lobectomy due to uncontrolled seizure disorder [37]. Seizure disorders can be highly debilitating for affected children and families. Seizures have been associated with regression in some cases [36], in addition to at least one report of mortality due to seizure-induced aspiration [2]. Seizures have not been intractable in most patients with PMS, but there is no information about whether certain anticonvulsant medications are more effective than others for these individuals. Overnight video-EEG is therefore recommended using the standard 10–20 system and 64 inputs of online spike and seizure detection programs for automated detection. Brief recordings may be inadequate to detect changes in some patients and sedation may be required to get reliable recordings. While an overnight EEG is not always needed to establish the diagnosis, it can be helpful in determining the type of seizures and assist in treatment decisions. There should also be a low threshold for repeating an EEG with signs of behavioral changes or regression, including loss of motor skills.

#### Brain imaging

Most descriptions of PMS report higher than expected rates of structural brain changes. Among the published case series, a total of 59 patients have had brain imaging, either through prospective assessment [30, 36, 52] or medical record review [34, 37]. Brain abnormalities were evident in 73% (43/59) of the cases, ranging from 44% (4/11) [34] to 100% (10/10) [52]. Changes included thinning or hypoplasia of the corpus callosum in 36% of cases (21/59); white matter changes such as delayed myelination, generalized white matter atrophy, and nonspecific white matter hyperintensities in 39% (23/59); ventricular dilatation in 32% (19/59), and interventricular, cerebellar, or temporal sylvian arachnoid cysts in 14% (8/59). The most recent prospective study specifically examined cerebellar malformations in ten patients with PMS using magnetic brain imaging (MRI) and found evidence of cerebellar vermis hypoplasia in six subjects, including an enlarged cisterna magna in five [52]. There have also been several patients with neurofibromatosis type 2 (NF2) reported in the literature who have ring chromosome 22 with NF2 features such as multiple intracranial meningiomas or vestibular schwannomas [53, 54]. Overall, there remains a paucity of data on the abnormal neural systems underlying this syndrome. Given the high prevalence of structural brain changes and that little is known about the neurobiology associated with PMS, brain imaging is recommended and routine monitoring may be indicated in some cases, depending on the abnormality. Specifically, new assessments of individuals affected with PMS should include structural MRI to assess morphology and to rule out the presence of cysts, and cerebellar, white matter, and corpus callosum abnormalities. It remains to be determined how frequently patients with PMS should have an MRI and how often monitoring should occur when indicated. While sedation is likely required to acquire meaningful imaging studies, the risks of the procedure must be balanced with the potential benefit, especially in the absence of clinical signs suggestive of structural pathology.

### Endocrinology

There are no systematic studies describing endocrine abnormalities in patients with PMS. There have been reports of both short stature [27, 29–31, 33, 37, 40] and accelerated growth [26, 27, 35, 41]. In a focused analysis of growth in 55 patients previously described by the Greenwood Genetic Center, Rollins [38] reported a larger than expected percentage of patients having short (11%) and tall (11%) stature (<5th percentile and >95th percentile, respectively), although the majority (78%) fell within the normal range. Short stature, in particular, has been specifically associated with ring chromosome 22 in some reports [30, 31, 33], although this is not a consistent finding in the literature [37]. Head circumference has also been specifically examined in several studies [31, 37–40], and individuals with PMS are more likely to have either microcephaly [(<3rd percentile) (6%–14% of cases, depending on the study) or macrocephaly [(>97th percentile)(7%–31% of cases)]. Hypothyroidism was present in 1 of 32 (3%) patients in one study [37] and 7 of 121 (6%) in another [39]. Hypertrichosis has also been reported in at least two subjects [37, 55]. Finally, there has been one case report of central diabetes insipidus in a 2-day old infant worth noting given what little is published about endocrine abnormalities in PMS [56].

Endocrine abnormalities should be considered in all children with PMS and assessed when clinically indicated. Proper nutrition would be indicated from body mass index (BMI) measurement and should be carefully assessed as children with PMS may have restricted diets and may ingest non-food items. Behavioral changes consistent with thyroid dysregulation should also be considered, including changes in activity level, cognition, or motor skills, and thyroid panels should be obtained to rule out hypothyroidism. As children get older, the possibility of early or late puberty should also be explored and monitored if necessary. Incomplete puberty such as premature adrenarche or premature thelarche should be examined as it would be for all children. Only precocious puberty has been reported in one study as occurring in 15 of 121 cases (12%) [39]. Menstruation can be particularly confusing and distressing for individuals with PMS, and hormonal cycling may contribute to behavioral changes in some children. Referral to endocrinology should be facilitated if specific endocrine abnormalities appear.

### Nephrology

Renal abnormalities are considered relatively common in PMS, with reports suggesting rates as high as 38% [37], but few studies have systematically examined the prevalence and none have used prospective methods. On comprehensive medical record review of 32 patients in one study [37], renal abnormalities included vesicoureteral reflux (13%), hydronephrosis (13%), renal agenesis (6%), dysplastic kidney (3%), and horseshoe kidneys and pyelectasis (3%). Another study documented renal problems in 39 of 148 cases (26%), including vesicoureteral reflux (14%), frequent urinary tract infections (8%), polycystic kidney (5%), duplicate kidney (1%), and dilated renal pelvis (5%) [39]. In the only other study to specifically report the prevalence of renal abnormalities, they were described in the context of genitourinary abnormalities in general, with 17% of patients affected with neonatal urinary infection and malformed clitoris, vesicoureteral reflux, unilateral multicystic kidney, and “partial renal failure” of unknown cause [31]. In at least one child, a unilateral multicystic kidney and Wilms’ tumor in the contralateral, unaffected kidney was detected using prenatal ultrasound [57]. As some of these genitourinary abnormalities may result in clinical disease and require treatment, it is recommended that all individuals with PMS have routine blood pressure measurement and renal and bladder sonography performed at the time of diagnosis and, if the sonogram is abnormal, be referred to a pediatric nephrologist or urologist for monitoring or treatment. Fetal sonography should not substitute for this study, as it is usually not performed after renal development is complete. Because sonography may be normal in cases of vesicoureteral reflux, urinary tract infection at a young age or recurrent urinary tract infections should also prompt nephrology or urology referral. Finally, for those with abnormal renal or bladder sonography or high blood pressure, urinalysis and kidney function blood tests (e.g., urea, creatinine, and electrolytes) should be obtained as the patient is being referred to expert care for additional guidance about specific ongoing monitoring recommendations.

### Cardiology

The prevalence of congenital heart defects (CHD) in PMS is highly variable. Phelan and McDermid (2011) report CHD in more than 25% of patients; the most common reported defects include tricuspid valve regurgitation, atrial septal defect, patent ductus arteriosus, and total anomalous pulmonary return [24, 41]. Soorya and colleagues [37] reported only one case with CHD (aortic regurgitation) in their series of 32 patients (3%), and Jeffries and colleagues [31] reported 4 of 30 (13%) cases to have CHD, two with patent ductus arteriosus and two with total anomalous pulmonary venous return. These reports are based on retrospective chart reviews and parent questionnaires, and there are no published data of systematic prospective evaluations for CHD in patients with PMS using standard of care methods. Because patients with PMS may have associated CHD that would necessitate medical and/or surgical intervention, we recommend a standard cardiac evaluation inclusive of a detailed exam, echocardiography, and electrocardiography as part of the initial evaluation in all patients with PMS.

### Gastroenterology

Gastrointestinal symptoms are common in PMS and include gastroesophageal reflux disease (GERD), constipation, and diarrhea [27, 37, 39, 41]. Rates are not consistently documented, but parent interview and medical record review of 32 cases suggest that GERD occurs in 44% of cases and constipation and/or diarrhea in 38% [37]. Another study reported similar rates, with 62 of 149 patients (42%) suffering from GERD and 11 of 27 (41%) from constipation [39]. Cyclic vomiting has also been described in several patients [25, 41]. Feeding difficulties are likewise common [30, 37] and may be related to low muscle tone. There have also been two reported cases of fulminant autoimmune hepatitis in girls with PMS [58, 59]. Gastrointestinal symptoms can be highly distressing and may manifest as appetite or behavioral changes in patients who cannot describe or identify their discomfort. Increased pain tolerance in PMS may further complicate the diagnosis of gastrointestinal disorders. Any change in appetite or behavior should raise suspicion of gastrointestinal distress. Dietary changes and bowel regimens should be considered with evidence of constipation and GERD may be treated empirically, depending on symptom patterns. Finally, chewing of non-food items and pica has been described in up to 50% of cases of PMS [25]. Severe chewing and pica may require the attention of a gastroenterologist and a referral for behavioral therapy.

### Primary care/developmental pediatrics

In addition to the multitude of specialized medical features characteristic of PMS, affected individuals are also prone to immune system dysfunction, including recurring ear and upper respiratory tract infections, seasonal allergies, food allergies, and asthma [30, 37, 39]. Repeated upper respiratory tract infections in PMS may be related, at least partially, to low muscle tone and subsequent problems with airway and sputum clearance. There is also evidence from animal studies that *SHANK3* protein may play a role in immune function, including immune cell signal transduction [60]. Children with PMS may suffer from immunological dysfunction based on case reports of autoimmune hepatitis [58, 59], atopic dermatitis [61], and recurring staphylococcus skin infections with a history of cellulitis [37]. Sarasua and colleagues [39] found cellulitis in 9 out of 137 (7%) patients. These conditions should be managed as they would in any other child, but recognizing them may be more challenging in PMS and warrant having a low threshold of suspicion for infections of all types. In addition, as with any child with developmental delays, early and aggressive referral to pediatric audiology and ophthalmology is important, especially as there have been case reports of PMS patients having hearing problems and vision problems including strabismus, myopia [27, 37, 39], and retinitis pigmentosa in one case [32]. Lymphedema has also been reported in the literature, including in up to 24% of cases in one study [39] and represents an especially troubling symptom for patients [27, 30, 35, 37, 39]. Routine management is warranted although benefits can be challenging to achieve. Compression boots, pneumatic pumps, or referral to vascular surgery may be considered in some cases. Thermoregulatory problems have also been described, including decreased perspiration and heat intolerance. Finally, dental abnormalities such as malocclusion, are common and can be quite severe in some cases [37, 41]. Orthodontic or surgical correction of malocclusion may be considered to reduce the risk of tooth decay and periodontal disease or relieve pressure on the temporomandibular joint.

## Conclusions

Phelan-McDermid syndrome is a complex and heterogeneous syndrome. While considered rare, the advent of advanced genetic analytic methods into clinical practice will likely identify more cases, and clinicians will require knowledge about appropriate assessment, and eventually treatment. CMA is the first tier in genetic analysis in addition to chromosome analysis to clarify the presence of ring chromosome 22 and translocations. Parents should also be tested for balanced rearrangement to assess recurrence risk in families. Sequencing of the *SHANK3* gene is necessary to identify mutations when CMA is unrevealing. Although *SHANK3* is understood to be the critical gene in the deletion syndrome, responsible for the core phenotypic features of PMS [2–4, 8, 62, 63], it is likely that other deleted genes contribute to the severity and additional phenotypic characteristics. Ongoing studies are focused on exploring genotype-phenotype correlations, and should relationships emerge, it may delineate a role for other genes and pathways in PMS. Further, genotype-phenotype associations may aid in medical monitoring and treatment planning if larger deletion sizes are associated with specific medical comorbidity or if greater impairments in language or motor skills might be predicted from genotype.

Point mutations disrupting only *SHANK3* have been described in the literature and result in a similar phenotype, including ASD and ID [2–4, 7, 37, 64, 65]. However, given the small number of patients identified to date, the full spectrum of phenotypic features has not clearly been defined and whether these patients are at the same increased risk of medical comorbidities as patients with deletions that encompass many genes in the region remains an active area of research. As whole exome and whole genome sequencing become widespread, these approaches will likely become the first line analyses in cases of unexplained developmental delay. It is estimated that the rates of mutation of *SHANK3* in ASD and developmental delay are in the same range as deletions [66], so PMS due to mutation in *SHANK3* will be increasingly identified.

A thorough history and physical examination is clearly the first step in evaluation. While there are several common medical and dysmorphic features, it does not appear that, as of yet, any features are specific for the syndrome. Neurological deficits are common in PMS. Motor deficits in particular may affect the child’s feeding and ability to thrive. The risk of seizures is greater in PMS and EEG is recommended, especially when there is evidence of skill regression. Given the prevalence of structural brain abnormalities in PMS, including arachnoid cysts, there should be a low threshold for performing brain imaging. The identification of specific brain abnormalities in PMS will aid in more thorough characterization and may provide a critical link between PMS and associated behavior. Furthermore, the creation of an accurate brain phenotype may be important for identifying biological markers of disease progression and possibly treatment response in the future. Endocrine, renal, cardiac, and gastrointestinal problems have all been reported in PMS and require assessment and monitoring. Other associated medical features include recurring infections that require standard management but may be more challenging to detect in PMS. Few primary care or developmental pediatricians have experience with PMS but will need to gain familiarity in order to play a critical role in coordinating care across subspecialties. It is equally crucial for the developmental pediatrician and other professionals involved in their care to support the family in advocating for the child’s educational and emotional needs by making sure educators and therapy providers understand the syndrome and its associated features. Appropriate referrals to specialists should be initiated early and interventions implemented intensively. Pediatric physiatry, physical therapy, occupational therapy, and orthopedics are among the specialists to explore the possibility of various interventions, including feeding therapy, orthotics, and bracing. All patients require referrals to specialized centers for cognitive, behavioral, and ASD evaluations (see Table 3).

**Table 3:** **Summary of clinical recommendations for assessment**

Medical specialty	Common clinical features	Assessments
Clinical genetics	Large fleshy hands	Dysmorphology exam
	Bulbous nose	
	Long eyelashes	
	Prominent/dysplastic ears	
	Hypoplastic/dysplastic nails	
	Dolichocephaly	
Molecular genetics		Chromosomal microarray
		Chromosome analysis (to identify ring chromosomes)
		Sanger or next generation sequencing (for mutations)
		Fluorescence *in situ* hybridization (to identify balanced rearrangements in parents)
Psychiatry	Autism spectrum disorder	Gold standard diagnostic assessments
Psychology	Aberrant behavior	Psychiatric evaluation
	Intellectual disability	Cognitive and adaptive behavior testing
	Absent or delayed speech	Speech and language evaluation
Neurology	Seizures	Overnight video electroencephalography
	Structural brain abnormalities	Brain imaging and head circumference monitoring
	Feeding difficulties	Feeding therapy evaluation
	Hypotonia	Occupational and physical therapy evaluations
	Motor skill deficits	
Endocrinology	Short/tall stature	Monitor height, weight, and body mass index
	Hypothyroidism	Metabolic work-up, including thyroid function
		Nutritional assessment
Nephrology	Vesicoureteral reflux	Renal and bladder ultrasonography
	Urinary tract infections	Voiding cystourethrogram
	Hydronephrosis	Monitoring of blood pressure
	Renal cysts, hypoplasia, or agenesis	
Cardiology	Congenital heart defects	Electrocardiography
		Echocardiography
Gastroenterology	Gastroesophageal reflux	Referral for dietary changes and/or medication
	Constipation/diarrhea	Bowel regimens
	Pica	Referral to behavioral therapy
Primary care/developmental pediatrics	Upper respiratory tract infections	Careful and consistent monitoring and management
	Recurring ear infections	Referral to otolaryngology, ophthalmology, physiatry, dental, and orthopedics
	Hearing and vision problems	
	Lymphedema	
	Dental problems	
	Decreased perspiration/heat intolerance	

Ongoing monitoring is crucial to track the disease course in PMS over time in order to better understand disease outcomes. There may be several developmental time points associated with increased risk of seizures, for example, and repeated assessments are necessary in suspected cases. In PMS, as patients age, they may be at increased risk for atypical bipolar disorder [29, 42, 43], and significant regression has also been reported in language, behavioral symptoms, motor skills, and self-help skills [2, 29, 37, 42, 43, 62]. Isolated reports have also emerged recently describing mortality associated with complications of PMS, including seizure-induced aspiration [2], renal failure in a patient with unilateral renal agenesis [62], and pneumonia [42]. Awareness of the full scope of medical comorbidity and aggressive monitoring will be critical to ensure quality of life and survival of patients with PMS.

In summary, comprehensive assessment and regular monitoring of patients with PMS across organ systems are necessary to clarify the extent and severity of the phenotype and to understand how PMS develops over time. Appropriate evaluations may also provide important information on targets for early intervention and disease prevention in order to further develop the best clinical practices. Establishing the natural history of PMS is also a important step toward designing effective clinical trials and may support the advancement of multiple therapeutic possibilities in the future.

## Abbreviations

ASD: autism spectrum disorder
BMI: body mass index
CMA: chromosomal microarray analysis
CHD: congenital heart defects
EEG: electroencephalography
FISH: fluorescence *in situ* hybridization
GERD: gastroesophageal reflux disease
ID: intellectual disability
PMS: Phelan-McDermid syndrome
MRI: magnetic resonance imaging.
